# Improving the treatment of infant pain

**DOI:** 10.1097/SPC.0000000000000270

**Published:** 2017-05-02

**Authors:** Fiona Moultrie, Rebeccah Slater, Caroline Hartley

**Affiliations:** Department of Paediatrics, University of Oxford, Oxford, UK

**Keywords:** analgesia, infant, nociception, pain

## Abstract

**Purpose of review:**

Pain management presents a major challenge in neonatal care. Newborn infants who require medical treatment can undergo frequent invasive procedures during a critical period of neurodevelopment. However, adequate analgesic provision is infrequently and inconsistently provided for acute noxious procedures because of limited and conflicting evidence regarding analgesic efficacy and safety of most commonly used pharmacological agents. Here, we review recent advances in the measurement of infant pain and discuss clinical trials that assess the efficacy of pharmacological analgesia in infants.

**Recent findings:**

Recently developed measures of noxious-evoked brain activity are sensitive to analgesic modulation, providing an objective quantitative outcome measure that can be used in clinical trials of analgesics.

**Summary:**

Noxious stimulation evokes changes in activity across all levels of the infant nervous system, including reflex activity, altered brain activity and behaviour, and long-lasting changes in infant physiological stability. A multimodal approach is needed if we are to identify efficacious and well tolerated analgesic treatments. Well designed clinical trials are urgently required to improve analgesic provision in the infant population.

## INTRODUCTION

Improved neonatal care has led to an increase in the number of invasive diagnostic tests and clinical interventions undertaken in infants. These procedures can be acutely painful, yet pharmacological analgesia is infrequently and inconsistently provided [1]. Chronic exposure to noxious stimuli during the preterm period is developmentally unexpected, and may drive changes in the maturation and organization of functional neural circuitry [2]. Given that infant pain is associated with short-term physiological instability [3] and long-term negative consequences, including changes in white matter microstructure [4,5] and altered cognitive development [5,6], effective analgesic provision is a clinical priority. Nevertheless, testing analgesic efficacy in infants requires a bespoke approach as verbal pain report clearly cannot be used and we cannot assume that analgesics used in adults and children will provide effective analgesia, because of differences in pharmacodynamics and pharmacokinetics [7].

Despite significant advances in our understanding of the neurobiology of pain, there remains a paucity of evidence-based analgesics available for use in infants [8,9^▪^]. Only in the past few decades have clinical trials been performed to assess the analgesic efficacy of pharmacological agents commonly used in neonatal care [10]. These trials use a range of validated and unvalidated pain scores, comprised of behavioural and physiological response variables, as endpoints to quantify pain experience [8,9^▪^]. They have yielded conflicting and controversial results, and positive effects have often been overshadowed by concerns over potential adverse drug effects [11]. This has led to extreme variation in analgesic practices both between and within countries [12^▪^–14^▪^]. A new approach is needed. Here, we emphasise that the assessment of analgesic efficacy in infants requires well designed age-appropriate clinical trials, using objective and sensitive endpoints assessed across multiple modalities to better quantify infant pain. We discuss evidence relating to the use of analgesics in neonatal practice and consider approaches to quantify analgesic efficacy using measurements of noxious-evoked change in infant physiology, behaviour and brain activity. 

**Box 1:**
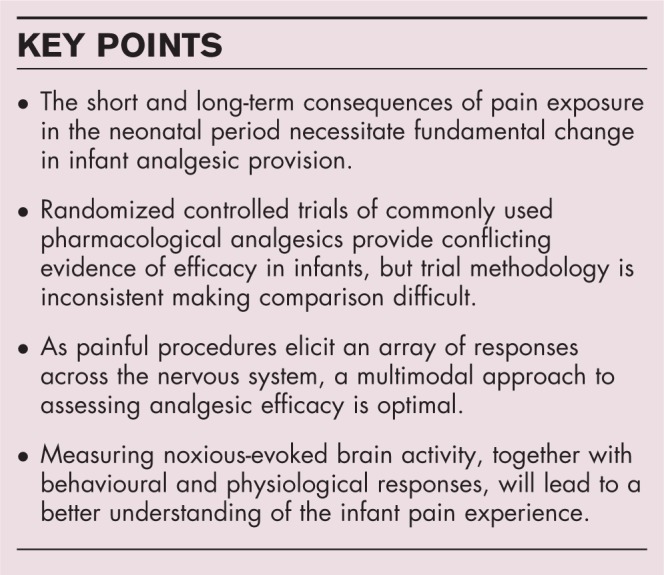
no caption available

## NOXIOUS-EVOKED ACTIVITY IN THE NEWBORN INFANT

Pain elicits an array of neurophysiological, behavioural, and physiological responses designed to protect the body from harm. When an infant undergoes a painful procedure, noxious information is transmitted from the periphery to the spinal cord via nociceptors, triggering a spinal reflex, which can be observed as bilateral limb withdrawal from the offending stimulus [15,16]. The noxious information passes to the brainstem, triggering physiological changes, as well as reflexive facial grimacing and vocalizations, which alert the caregiver. It is then transmitted via the thalamus to various cortical brain regions [17^▪^,^18^], which in adults, are thought to encode the sensory and emotional aspects of pain [19]. Cortical and subcortical brain regions also have a top–down modulatory effect on the nociceptive signal [20], which changes with development [21,22]. Physiological stability can be disrupted for several hours after the nociceptive event, with increased prevalence of episodes of tachycardia or bradycardia, oxygen desaturations, and apnoeas [23,24]. Up until recently, much of our understanding of clinical pain assessment and management in infants has been based upon the scoring of noxious-evoked behaviour and physiological responses [10]. These pioneering studies have been highly influential in raising the profile of infant pain and have provided good evidence for the use of non-pharmacological comfort techniques [25,26,27^▪^]. However, when these measures have been used to test pharmacological agents they have yielded inconsistent results, and have contributed to a continual state of equipoise for most analgesics.

## SEARCHING FOR ANALGESIC EFFICACY: MORPHINE – A CLASSIC EXAMPLE

Analgesic agents have mostly been introduced into neonatal practice unconventionally, with limited understanding of their pharmacokinetics and pharmacodynamics, and without clinical trials establishing their efficacy [10]. Doses of many commonly used drugs are often extrapolated from adult and paediatric regimens [10]. Morphine, the archetypal opioid, is the most frequently used analgesic in infants [13^▪^] and while it has been shown to reduce physiological instability and hormonal stress responses [28–31], and improve ventilator synchrony [32] in premature infants, its analgesic efficacy remains controversial [13^▪^,^33^].

The efficacy of intravenous morphine has been studied using various behavioural pain scores across a variety of acute noxious procedures, including heel lancing [34,35], tracheal suctioning [28,33,36], elective intubation [37], and peripheral central venous cannulation [38]. Studies have yielded contradictory results, and differences in study methodologies, drug dosages, heterogeneity of outcome measures, and clinical procedures, as well as administration of ‘rescue’ opioid boluses to control groups, have made interpretation of the evidence challenging. Three large randomized placebo-controlled trials have tested the analgesic efficacy of intravenous morphine in the context of tracheal suctioning [28,33,36]. Although two of these studies appeared to demonstrate a significant reduction in a well validated pain score, the Premature Infant Pain Profile [28,33], results were not consistent across time points in one of the studies, and the statistically significant result did not equate to clinical significance [33]. A third study [36], found no significant effect of morphine infusion on three behavioural pain scores assessed before, during, and after endotracheal suctioning. This study, however, followed the principle of intention-to-treat and permitted the administration of open-label morphine at the discretion of attending physicians. In total, 40% of the placebo group received open-label morphine as the infants were considered to be ‘uncomfortable’ and requiring additional pain relief. The authors reported poor concordance between pain scores and acknowledged the potential lack of sensitivity and specificity of pain scoring methods. Meta-analysis unsurprisingly revealed significant heterogeneity, and failed to identify a significant effect of morphine [8].

**FIGURE 1:**
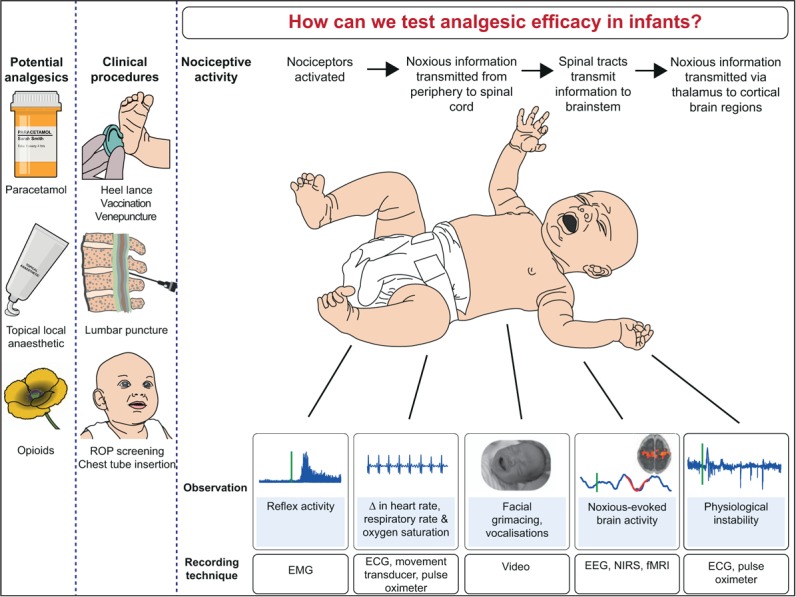
Measuring analgesic efficacy in infants. EEG, electroencephalography; EMG, electromyography; fMRI, functional MRI; NIRS, near-infrared spectroscopy; ROP, retinopathy of prematurity.

Without sensitive and specific endpoints the analgesic efficacy of a drug cannot be appropriately evaluated. Can we really expect brief behavioural, physiological, or neurophysiological snapshots in isolation to provide compelling evidence to convince the neonatal community to administer an analgesic like morphine? Particularly in light of potential side-effects and long-term consequences [11], more detailed evidence of analgesic efficacy is clearly required. As pain elicits a wide spectrum of responses, a multimodal approach incorporating noxious-evoked brain and spinal activity, as well as behavioural and physiological measures, will provide a more complete understanding of infant pain (Fig. 1).

## THE MEASUREMENT OF NOXIOUS-EVOKED BRAIN ACTIVITY IN INFANTS

Noxious-evoked brain activity was first recorded in newborn infants approximately 10 years ago [39–41], and since then it has been shown to be graded with stimulus intensity [16], dependent on gestational age [21,39,42,43], and is elicited across a network of brain regions which are similar to those activated in adults [17^▪^]. Although noxious-evoked brain activity, spinally mediated reflex withdrawal, and pain-related facial activity are relatively well correlated [16,44^▪^,^45^], noxious information can be transmitted to the cortex without producing observable behavioural changes [44^▪^,^45^]. Therefore, infants with low pain scores based on behavioural assessment alone may not be pain free [45]. Potential discordance between brain activity and various noxious-evoked patterns of physiology and behaviour was exemplified by a blinded randomized controlled trial investigating the analgesic efficacy of sucrose, a popular non-pharmacological analgesic [46]. Although a Cochrane review of 74 studies across a total of more than 7000 infants suggests that sucrose is effective for short procedures [27^▪^], noxious-evoked brain activity and reflex withdrawal to a heel lance are not altered by sucrose administration [46]. Measures of noxious-evoked brain and spinal cord activity appear more sensitive than facial expression change [16,47], and therefore lack of sensitivity of the noxious-evoked brain activity is unlikely to be the cause of the discordance between these measures. Analgesics work by modulating nociceptive input to the brain and these results suggest that although sucrose may dampen behavioural signs of distress it may not provide analgesia, as it is not altering the noxious input transmitted to the brain.

This year an electroencephalography (EEG)-based template has been developed that can objectively quantify the magnitude of brain activity evoked by noxious stimulation [44^▪^]. The validity of the template was tested across four independent samples of infants, ranging from 34 to 43 weeks’ gestation. It robustly quantifies the nociceptive afferent brain activity, and allows direct comparison of noxious-evoked activity across different infant groups. Although the infant pain experience clearly cannot be solely represented by a brief pattern of electrical activity recorded at a single electrode site within 1 s of a noxious event, this method does provide sensitive quantification of noxious input reaching the brain. Importantly, it also provides a standardized approach for the measurement of analgesic efficacy, which can be used in clinical trials of analgesics [48,49^▪^], and potentially in dose-finding studies. Topical application of tetracaine, a potent local anaesthetic, significantly reduces the magnitude of noxious-evoked brain activity quantified by the template [44^▪^]. This is a critical demonstration that noxious-evoked brain activity is sensitive to analgesic modulation.

Similarly to morphine, behavioural pain score studies have provided inadequate evidence to establish the analgesic efficacy of topical local anaesthetics for needle-related pain in infants [9^▪^]. Although multiple studies have demonstrated that topical local anaesthetics can reduce clinical pain scores [50–54,55^▪^], there are numerous conflicting reports that challenge these observations across a range of clinical procedures, including heel lancing [56–60], venepuncture [61], and intramuscular injections [62,63]. In part these mixed results may be because of the heterogeneity across the study designs, such as the choice of local anaesthetic, length of application, pain assessment endpoints, age group of the infants, and low study participant number. Nevertheless, it is also plausible that the reported lack of analgesic efficacy may arise because the behavioural measures used to assess pain are not sensitive enough, and may be confounded by distress caused by non-noxious aspects of the procedures, such as the need to physically restrain the infant. Although all efforts should, of course, be made to limit infant distress as well as pain, the challenge of disambiguating these responses can make it difficult to measure the antinociceptive properties of pharmacological analgesic agents. Using a multimodal approach in clinical trials of analgesics that includes measures of noxious-evoked brain activity may help address these challenges.

## IS MORPHINE AN EFFECTIVE ANALGESIC FOR PROCEDURAL PAIN IN INFANTS?

The Procedural Pain in Premature Infants (POPPI) trial is a recent example of a study where multidimensional measures could provide a better understanding of the effect of potent analgesic compounds [48,49^▪^]. The POPPI trial is a blinded randomized controlled trial currently in progress, which aims to establish whether morphine provides effective analgesia for acute pain in prematurely born infants. Infants are randomized to receive oral morphine or placebo approximately 1 hour prior to an invasive eye examination for retinopathy of prematurity (ROP screening) coupled with a routine heel lance. Noxious-evoked changes in brain activity, spinal cord activity, heart rate, oxygen saturation, and behaviour are recorded during these painful procedures, as are longer term changes in physiological stability. In addition, drug safety is assessed for 24 hours after drug administration. If the results of the trial show that the administration of a single oral bolus dose of morphine prior to clinical heel lancing and ROP screening decreases noxious-evoked brain activity, reduces clinical pain scores, and prevents the physiological instability reported to occur in the 24 hour period after ROP screening, there would be a strong rationale for the use of morphine in clinical practice.

## DEVELOPING BRAIN ACTIVITY MEASURES TO IMPROVE ANALGESIC DRUG DISCOVERY

Although the EEG template [44^▪^] provides an opportunity to quantify afferent nociceptive input and compare this activity across multiple studies, there are several inherent limitations currently associated with this technique. At present, it is only validated in a limited age range of infants, and has only been characterized in response to brief experimental noxious stimulation and clinical heel lance. Nevertheless, similar patterns of noxious-evoked activity have been recorded in response to vaccination [47], suggesting that the template could be developed to have more wide-reaching applicability. Importantly, this template is not a ‘gold-standard’ measure of brain activity. It does not preclude the exploration of other features of noxious-evoked brain activity, such as analysis in the time-frequency domain [64^▪^] that will no doubt lead to further new insights. Integrating EEG responses with measures of haemodynamic activity using near-infrared spectroscopy (which can be done simultaneously at the cot side) [65] will also provide new understanding, for example, with regards to the development of neurovascular coupling [66]. Moreover, functional MRI (fMRI), which has been extensively used to understand the brain activity and connectivity underlying the sensory, cognitive, and emotional aspects of the adult pain experience [19,20], may provide important additional information in infants [17^▪^,^18^]. Machine learning techniques have been used to identify sensitive and specific fMRI neural signatures of pain in adults [67], and key potential applications include their use as surrogate biomarkers for drug discovery and for targeted analgesic treatments [67,68]. Given the inherent lack of infant language, characterizing the neural representation of noxious-evoked brain activity in the infant could be one of the most important applications for these techniques. There is so much that we can learn from the adult pain imaging literature, but also much that infant pain research can contribute to the understanding of the long-term development of adult pain. Identifying analgesics that can modulate noxious-evoked brain activity in infants is important, but furthering this work such that we know how analgesics impact brain activity across multiple brain regions is crucial if we are to understand mechanisms of action and improve analgesic efficacy.

## CONCLUSION

Pain is a complex sensory and emotional perception. Painful procedures trigger an array of responses across the body, which include reflexes, facial grimacing, changes in cortical activity, and disruption of physiological stability. Given the absolute requirement to quantify infant pain based on measurable changes in noxious-evoked activity, it is evident that infant pain cannot be interpreted by considering isolated measures; a composite assessment is required. An effective analgesic should ultimately reduce the transmission of noxious input to the brain and result in a reduction in observed behavioural distress and subsequent signs of physiological instability. It remains to be seen whether currently used analgesics can satisfy the conditions of this multimodal approach. Well designed clinical trials are urgently required to improve the provision of effective analgesia in this unique patient group.

## Acknowledgements

*We would like to thank Charlotte Dyson and Ravi Poorun for help preparingFig. 1*.

### Financial support and sponsorship

The work was supported by the Wellcome Trust [095802] and National Institute of Health Research (NIHR) [14/187/01]. NIHR funding is provided through the Efficacy and Mechanism Evaluation (EME) Programme, an MRC and NIHR partnership [14/187/01]. F.M. is a doctoral training fellow supported by the Wellcome Trust [102176] and NIHR Biomedical Research Centre, based at Oxford University Hospitals NHS Trust, Oxford.

### Conflicts of interest

There are no conflicts of interest.

## REFERENCES AND RECOMMENDED READING

Papers of particular interest, published within the annual period of review, have been highlighted as:

▪ of special interest
▪▪ of outstanding interest

## References

[1] Roofthooft DW Simons SH Anand KJ Eight years later, are we still hurting newborn infants? Neonatology 2014; 105:218–226. 24503902 10.1159/000357207

[2] Fitzgerald M The development of nociceptive circuits. Nat Rev Neurosci 2005; 6:507–520. 15995722 10.1038/nrn1701

[3] Morison SJ Grunau RE Oberlander TF Whitfield MF Relations between behavioral and cardiac autonomic reactivity to acute pain in preterm neonates. Clin J Pain 2001; 17:350–358. 11783816 10.1097/00002508-200112000-00010 PMC1852479

[4] Brummelte S Grunau RE Chau V Procedural pain and brain development in premature newborns. Ann Neurol 2012; 71:385–396. 22374882 10.1002/ana.22267 PMC3760843

[5] Vinall J Miller SP Bjornson BH Invasive procedures in preterm children: brain and cognitive development at school age. Pediatrics 2014; 133:412–421. 24534406 10.1542/peds.2013-1863 PMC3934331

[6] Doesburg SM Chau CM Cheung TP Neonatal pain-related stress, functional cortical activity and visual-perceptual abilities in school-age children born at extremely low gestational age. Pain 2013; 154:1946–1952. 23711638 10.1016/j.pain.2013.04.009 PMC3778166

[7] Kearns GL Abdel-Rahman SM Alander SW Developmental pharmacology: drug disposition, action, and therapy in infants and children. N Engl J Med 2003; 349:1157–1167. 13679531 10.1056/NEJMra035092

[8] Bellu R de Waal K Zanini R Opioids for neonates receiving mechanical ventilation: a systematic review and meta-analysis. Arch Dis Child Fetal Neonatal Ed 2010; 95:F241–F251. 19531519 10.1136/adc.2008.150318

[9▪] Foster JP Taylor C Spence K Topical anaesthesia for needle-related pain in newborn infants. Cochrane Database Syst Rev 2017; 2:CD010331. 28160271 10.1002/14651858.CD010331.pub2 PMC6464546

[10] Baarslag MA Allegaert K Van Den Anker JN Paracetamol and morphine for infant and neonatal pain; still a long way to go? Expert Rev Clin Pharmacol 2017; 10:111–126. 27785937 10.1080/17512433.2017.1254040

[11] Smits A van den Anker JN Allegaert K Clinical pharmacology of analgosedatives in neonates: ways to improve their safe and effective use. J Pharm Pharmacol 2017; 69:350–360. 27364566 10.1111/jphp.12599 PMC5201450

[12▪] Borenstein-Levin L Synnes A Grunau RE Narcotics and sedative use in preterm neonates. J Pediatr 2017; 180:92.e1–98.e1. 27614931 10.1016/j.jpeds.2016.08.031

[13▪] Carbajal R Eriksson M Courtois E Sedation and analgesia practices in neonatal intensive care units (EUROPAIN): results from a prospective cohort study. Lancet Respir Med 2015; 3:796–812. 26420017 10.1016/S2213-2600(15)00331-8

[14▪] Zimmerman KO Smith PB Benjamin DK Sedation, analgesia, and paralysis during mechanical ventilation of premature infants. J Pediatr 2017; 180:99.e1–104.e1. 27522446 10.1016/j.jpeds.2016.07.001 PMC5183489

[15] Cornelissen L Fabrizi L Patten D Postnatal temporal, spatial and modality tuning of nociceptive cutaneous flexion reflexes in human infants. PLoS One 2013; 8:e76470. 24124564 10.1371/journal.pone.0076470 PMC3790695

[16] Hartley C Goksan S Poorun R The relationship between nociceptive brain activity, spinal reflex withdrawal and behaviour in newborn infants. Sci Rep 2015; 5:12519. 26228435 10.1038/srep12519 PMC4521152

[17▪] Goksan S Hartley C Emery F fMRI reveals neural activity overlap between adult and infant pain. ELife 2015; 4: 10.7554/eLife.06356 PMC4402596 25895592

[18] Williams G Fabrizi L Meek J Functional magnetic resonance imaging can be used to explore tactile and nociceptive processing in the infant brain. Acta Paediatr 2015; 104:158–166. 25358870 10.1111/apa.12848 PMC4463763

[19] Apkarian AV Bushnell MC Treede RD Zubieta JK Human brain mechanisms of pain perception and regulation in health and disease. Eur J Pain 2005; 9:463–484. 15979027 10.1016/j.ejpain.2004.11.001

[20] Bingel U Tracey I Imaging CNS modulation of pain in humans. Physiology 2008; 23:371–380. 19074744 10.1152/physiol.00024.2008

[21] Hartley C Moultrie F Gursul D Changing balance of spinal cord excitability and nociceptive brain activity in early human development. Curr Biol 2016; 26:1998–2002. 27374336 10.1016/j.cub.2016.05.054 PMC4985558

[22] Hathway GJ Koch S Low L Fitzgerald M The changing balance of brainstem-spinal cord modulation of pain processing over the first weeks of rat postnatal life. J Physiol 2009; 587:2927–2935. 19403624 10.1113/jphysiol.2008.168013 PMC2718251

[23] Belda S Pallas CR De la Cruz J Tejada P Screening for retinopathy of prematurity: is it painful? Biol Neonate 2004; 86:195–200. 15240989 10.1159/000079542

[24] Mitchell A Stevens B Mungan N Analgesic effects of oral sucrose and pacifier during eye examinations for retinopathy of prematurity. Pain Manag Nurs 2004; 5:160–168. 15616486 10.1016/j.pmn.2004.06.001

[25] Harrison D Reszel J Bueno M Breastfeeding for procedural pain in infants beyond the neonatal period. Cochrane Database Syst Rev 2016; 10:CD011248. 27792244 10.1002/14651858.CD011248.pub2 PMC6461192

[26] Johnston C Campbell-Yeo M Disher T Skin-to-skin care for procedural pain in neonates. Cochrane Database Syst Rev 2017; 2:CD008435. 28205208 10.1002/14651858.CD008435.pub3 PMC6464258

[27▪] Stevens B Yamada J Ohlsson A Sucrose for analgesia in newborn infants undergoing painful procedures. Cochrane Database Syst Rev 2016; 7:CD001069. 27420164 10.1002/14651858.CD001069.pub5 PMC6457867

[28] Anand KJ Barton BA McIntosh N Analgesia and sedation in preterm neonates who require ventilatory support: results from the NOPAIN trial. Neonatal Outcome and Prolonged Analgesia in Neonates. Arch Pediatr Adolesc Med 1999; 153:331–338. 10201714 10.1001/archpedi.153.4.331

[29] Quinn MW Otoo F Rushforth JA Effect of morphine and pancuronium on the stress response in ventilated preterm infants. Early Hum Dev 1992; 30:241–248. 1468386 10.1016/0378-3782(92)90073-p

[30] Quinn MW Wild J Dean HG Randomised double-blind controlled trial of effect of morphine on catecholamine concentrations in ventilated preterm babies. Lancet 1993; 342:324–327. 8101584 10.1016/0140-6736(93)91472-x

[31] Simons SH van Dijk M van Lingen RA Randomised controlled trial evaluating effects of morphine on plasma adrenaline/noradrenaline concentrations in newborns. Arch Dis Child Fetal Neonatal Ed 2005; 90:F36–F40. 15613571 10.1136/adc.2003.046425 PMC1721820

[32] Dyke MP Kohan R Evans S Morphine increases synchronous ventilation in preterm infants. J Paediatr Child Health 1995; 31:176–179. 7669374 10.1111/j.1440-1754.1995.tb00780.x

[33] Anand KJ Hall RW Desai N Effects of morphine analgesia in ventilated preterm neonates: primary outcomes from the NEOPAIN randomised trial. Lancet 2004; 363:1673–1682. 15158628 10.1016/S0140-6736(04)16251-X

[34] Carbajal R Lenclen R Jugie M Morphine does not provide adequate analgesia for acute procedural pain among preterm neonates. Pediatrics 2005; 115:1494–1500. 15930209 10.1542/peds.2004-1425

[35] Scott CS Riggs KW Ling EW Morphine pharmacokinetics and pain assessment in premature newborns. J Pediatr 1999; 135:423–429. 10518075 10.1016/s0022-3476(99)70163-0

[36] Simons SH van Dijk M van Lingen RA Routine morphine infusion in preterm newborns who received ventilatory support: a randomized controlled trial. JAMA 2003; 290:2419–2427. 14612478 10.1001/jama.290.18.2419

[37] Lemyre B Doucette J Kalyn A Morphine for elective endotracheal intubation in neonates: a randomized trial [ISRCTN43546373]. BMC Pediatr 2004; 4:20. 15461825 10.1186/1471-2431-4-20 PMC524358

[38] Taddio A Lee C Yip A Intravenous morphine and topical tetracaine for treatment of pain in preterm neonates undergoing central line placement. JAMA 2006; 295:793–800. 16478902 10.1001/jama.295.7.793

[39] Slater R Cantarella A Gallella S Cortical pain responses in human infants. J Neurosci 2006; 26:3662–3666. 16597720 10.1523/JNEUROSCI.0348-06.2006 PMC6674141

[40] Slater R Worley A Fabrizi L Evoked potentials generated by noxious stimulation in the human infant brain. Eur J Pain 2010; 14:321–326. 19481484 10.1016/j.ejpain.2009.05.005

[41] Bartocci M Bergqvist LL Lagercrantz H Anand KJ Pain activates cortical areas in the preterm newborn brain. Pain 2006; 122:109–117. 16530965 10.1016/j.pain.2006.01.015

[42] Fabrizi L Slater R Worley A A shift in sensory processing that enables the developing human brain to discriminate touch from pain. Curr Biol 2011; 21:1552–1558. 21906948 10.1016/j.cub.2011.08.010 PMC3191265

[43] Bembich S Marrazzo F Barini A The cortical response to a noxious procedure changes over time in preterm infants. Pain 2016; 157:1979–1987. 27152689 10.1097/j.pain.0000000000000605

[44▪] Hartley C Duff E Green G Nociceptive brain activity as a measure of analgesic efficacy in infants. Sci Transl Med 2017. 10.1126/scitranslmed.aah6122 PMC5884430 28469039

[45] Slater R Cantarella A Franck L How well do clinical pain assessment tools reflect pain in infants? PLoS Med 2008; 5:e129. 18578562 10.1371/journal.pmed.0050129 PMC2504041

[46] Slater R Cornelissen L Fabrizi L Oral sucrose as an analgesic drug for procedural pain in newborn infants: a randomised controlled trial. Lancet 2010; 376:1225–1232. 20817247 10.1016/S0140-6736(10)61303-7 PMC2958259

[47] Verriotis M Fabrizi L Lee A Cortical activity evoked by inoculation needle prick in infants up to one-year old. Pain 2015; 156:222–230. 25599443 10.1097/01.j.pain.0000460302.56325.0c PMC4309489

[48] Hartley C Moultrie F Juszczak E Protocol 15PRT/5747: a blinded randomised placebo-controlled trial investigating the efficacy of morphine analgesia for procedural pain in infants. Lancet 2016; www.thelancet.com/protocol-reviews/15PRT-5747 10.12688/wellcomeopenres.10005.2 PMC5218543 28066825

[49▪] Slater R Hartley C Moultrie F A blinded randomised placebo-controlled trial investigating the efficacy of morphine analgesia for procedural pain in infants: trial protocol. Wellcome Open Res 2016; 1:7. 28066825 10.12688/wellcomeopenres.10005.2 PMC5218543

[50] Garcia OC Reichberg S Brion LP Schulman M Topical anesthesia for line insertion in very low birth weight infants. J Perinatol 1997; 17:477–480. 9447537

[51] Jain A Rutter N Does topical amethocaine gel reduce the pain of venepuncture in newborn infants? A randomised double blind controlled trial. Arch Dis Child Fetal Neonatal Ed 2000; 83:F207–F210. 11040170 10.1136/fn.83.3.F207 PMC1721170

[52] Kaur G Gupta P Kumar A A randomized trial of eutectic mixture of local anesthetics during lumbar puncture in newborns. Arch Pediatr Adolesc Med 2003; 157:1065–1070. 14609894 10.1001/archpedi.157.11.1065

[53] Larsson BA Tannfeldt G Lagercrantz H Olsson GL Alleviation of the pain of venepuncture in neonates. Acta Paediatr 1998; 87:774–779. 9722252 10.1080/080352598750013879

[54] Moore J No more tears: a randomized controlled double-blind trial of amethocaine gel vs. placebo in the management of procedural pain in neonates. J Adv Nurs 2001; 34:475–482. 11380714 10.1046/j.1365-2648.2001.01776.x

[55▪] Taddio A Pillai Riddell R Ipp M Relative effectiveness of additive pain interventions during vaccination in infants. CMAJ 2017; 189:E227–E234. 27956393 10.1503/cmaj.160542 PMC5305402

[56] Bonetto G Salvatico E Varela N Pain prevention in term neonates: randomized trial for three methods. Arch Argent Pediatr 2008; 106:392–396. 19030637 10.1590/S0325-00752008000500004

[57] Jain A Rutter N Ratnayaka M Topical amethocaine gel for pain relief of heel prick blood sampling: a randomised double blind controlled trial. Arch Dis Child Fetal Neonatal Ed 2001; 84:F56–F59. 11124928 10.1136/fn.84.1.F56 PMC1721197

[58] Larsson BA Jylli L Lagercrantz H Olsson GL Does a local anaesthetic cream (EMLA) alleviate pain from heel-lancing in neonates? Acta Anaesthesiol Scand 1995; 39:1028–1031. 8607303 10.1111/j.1399-6576.1995.tb04223.x

[59] McIntosh N van Veen L Brameyer H Alleviation of the pain of heel prick in preterm infants. Arch Dis Child Fetal Neonatal Ed 1994; 70:F177–F181. 8198410 10.1136/fn.70.3.f177 PMC1061036

[60] Stevens B Johnston C Taddio A Management of pain from heel lance with lidocaine-prilocaine (EMLA) cream: is it safe and efficacious in preterm infants? J Dev Behav Pediatr 1999; 20:216–221. 10475595 10.1097/00004703-199908000-00003

[61] Lemyre B Hogan DL Gaboury I How effective is tetracaine 4% gel, before a venipuncture, in reducing procedural pain in infants: a randomized double-blind placebo controlled trial. BMC Pediatr 2007; 7:7. 17288611 10.1186/1471-2431-7-7 PMC1800845

[62] Newby BD Faschoway GD Soukoroff CI Tetracaine (ametop) compared to placebo for reducing pain associated with intramuscular injection of palivizumab (synagis). J Pediatr Nurs 2009; 24:529–533. 19931151 10.1016/j.pedn.2009.05.007

[63] Shah VS Taddio A Hancock R Topical amethocaine gel 4% for intramuscular injection in term neonates: a double-blind, placebo-controlled, randomized trial. Clin Ther 2008; 30:166–174. 18343253 10.1016/j.clinthera.2008.01.018

[64▪] Fabrizi L Verriotis M Williams G Encoding of mechanical nociception differs in the adult and infant brain. Sci Rep 2016; 6:28642. 27345331 10.1038/srep28642 PMC4921818

[65] Verriotis M Fabrizi L Lee A Mapping cortical responses to somatosensory stimuli in human infants with simultaneous near-infrared spectroscopy and event-related potential recording. eNeuro 2016; 3: https://www.ncbi.nlm.nih.gov/pubmed/27200413 10.1523/ENEURO.0026-16.2016 PMC4867026 27200413

[66] Arichi T Fagiolo G Varela M Development of BOLD signal hemodynamic responses in the human brain. Neuroimage 2012; 63:663–673. 22776460 10.1016/j.neuroimage.2012.06.054 PMC3459097

[67] Wager TD Atlas LY Lindquist MA An fMRI-based neurologic signature of physical pain. N Engl J Med 2013; 368:1388–1397. 23574118 10.1056/NEJMoa1204471 PMC3691100

[68] Duff EP Vennart W Wise RG Learning to identify CNS drug action and efficacy using multistudy fMRI data. Sci Transl Med 2015; 7:274ra16. 10.1126/scitranslmed.3008438 25673761

